# My covid-19 experience: picking up the pieces

**DOI:** 10.4314/ahs.v20i4.4

**Published:** 2020-12

**Authors:** Oliver Ombeva Malande

**Affiliations:** 1 East Africa Centre for Vaccines and Immunization (ECAVI); 2 Egerton University, Department of Paediatrics & Child Health; 3 Makerere University, Department of Paediatrics & Child Health

## Introduction

Around December 2019, an increasing number of pneumonia cases of unknown origin were reported in the Chinese city of Wuhan 1–5. Scientists later found this to be caused by a virus, that was identified as an enveloped RNA betacoronavirus, which later was named by World Health Organization (WHO) as 2019-nCoV with the associated illness as COVID-19. As I write this memoir, almost a year later, today (29^th^ November 2020) a total of 62,729,958 COVID-19 cases worldwide have been confirmed, out of whom 1,460,993 have died^6^. Early last month, I was added to this statistic. Some people question the existence of COVID-19 both as a disease and as an entity. Their argument has been that they do not know of anyone who is close or related to them who has suffered from the COVID-19 infection. It is understandable anyway, especially knowing that this viral infection has not been with us for too long, hardly a year, yet it has caused untold suffering, loss, pain, and long-term disabilities to many. I am a recovering COVID-19 patient, and I take comfort in the knowledge that sharing my story will encourage many other affected people to come out and share their stories as well. I am particularly concerned about health care workers, who keep quiet and suffer in silence. I know so many of my colleagues across East Africa who are quietly and silently suffering from COVID-19, and won't mention it to anyone for all sorts of fears, including stigma. Others are at different levels of sorrow, especially those that have suffered loss of a loved one accompanying this unfortunate experience.

My story I think is similar to that of many health workers affected by COVID-19. It so happened that I was called to review a child who was not responding to antibiotic cover for pneumonia. I went to the facility, and I must admit that the personal protective equipment (PPE) I used was not optimum. I spent a significant time of exposure with this child and her mother, who was also sickly and coughing frequently. After about an hour, I considered a COVID-19 PCR test for the child and the mother. Early the following day, the results came and they were both positive for SARS-COV2. I knew that I had been significantly exposed and ran a real risk of contracting corona virus. About four days later, I developed persistent headache unresponsive to paracetamol and flu-like symptoms. I decide to get tested, for corona virus, and the results confirmed it, I was COVID-19 PCR positive. By the time I got the results, I had red itchy eyes, sore throat, severe muscle aches, severe joint pains and abdominal pain. These pains worsened to the point where no oral medication for pain control could relieve it. My attending physician decided that I needed a stronger approach to pain management. So, I was put on injectable diclofenac 75mg IM 8–12 hourly, with other medications that included one zinc tablet daily, one ascorbic acid. I also received daily azithromycin and hydroxychloroquine (HCQ) for 5 days. I do understand the reservations many people have against HCQ, especially with the solidarity trial results^7^ that showed none of the studied drugs including HCQ showed any benefits, but for my case I had already played this scenario in my head several times and was ready to take HCQ if and when I get tested positive for SARS-COV2. I cannot for certain say it helped me.

I have never experienced the kind of pain and myalgia I suffered during the illness, it was severe and debilitating. I also had extreme fatigue, and could hardly lift a twenty litre jerrican of water. Air hunger and breathlessness did occur, and it would appear to me that they are features that occur at night mostly. On two separate nights in my first 5 – 10 days after COVID diagnosis, I suffered from episodes of breathlessness and air hunger, which were as if I was drowning. This puzzled me, especially knowing that I am young, fit, and have no comorbidities whatsoever. I exercise and generally watch my diet and health carefully. Why would I have episodes of air hunger? These happened in the night, at around 2:00 am. At the time, I also experienced weakness, tiredness and drenching night sweats, alongside the irritating persistent dry cough, headache that was almost splitting my head into two – that only made the muscle and joint pains even worse. These hypoxic low oxygen nights were scary, and I used maneuvers like squatting, lying in recovery position and changing from supine to prone position to slowly regain composure and stability. I tried to remain as calm as I could, I reasoned that what will be will be, but I will go down fighting. All I needed was half a chance at survival, and I would take it… a rub of the green of some sort. As I slowly recovered from the hypoxic spells, I reached out for my pulse oximeter and what a relief when I found I was averaging 94–95 % oxygen saturation in room air. The COVID-19 infection pushes you to the limit, and at some point, you will have to summon all your energy and inner strength to stay afloat. It is that critical moment when the rubber meets the road, and when you have to look at yourself and speak to yourself and say; I am ready for this fight, I will give as much as I get, and I will not drown, my head will remain above the water. The feeling like you are ‘drowning’ and experiencing ‘air hunger’ is very scary, even for experienced swimmers, it is staring at death itself, walking through the valley of the shadow of death literally. When and if that happens, be ready to look the devil in the eye and fight the demons. You've got to give yourself a chance to survive, in any case, if you don't buy a ticket, you can't win a raffle. It is about survival. COVID is a war, and any one who receives a positive result must approach it with no other mindset, than to admit that it is a war, and the desired outcome is only one: SURVIVAL.

Currently, I belong to a large group of medical workers recovering from COVID-19. I asked them to share their commonest symptoms of COVID-19, and these are some sampled responses:

**Respondent 1:** severe fatigue and myalgia, throbbing headache, fever, nausea, chills.

**Respondent 2:** headache on and off, muscle aches especially in the calf muscles when walking, fatigue, sore throat.

**Respondent 3:** my symptoms were atypical – diarrhea, an extreme dry mouth (I kept checking my sugars though am not diabetic), a continuous bitter taste in the mouth, severe chest pains, fast heart beats, and insomnia - when I do get sleep, I am woken up by nightmares.

**Respondent 4:** I started with fevers, body ache, excessive fatigue slight dry cough, nasal stuffiness and excessive sweating. Week 2 had loss of taste and smell, which subsided after a week. Week 3 started fever headache and nausea and vomiting. Week 4: slight headache that deteriorated as time went by, diarrhea, no sense of smell or taste, general malaise, nausea, loss of appetite, breathlessness.

**Respondent 5:** headache, chills, myalgia, fatigue, sore throat, chest pains, loss of smell.

**Respondent 6:** I had loss of taste and smell, currently week 2, I have excessive sweating, body aches & burning body sensations; extreme fatigue all the time, mental fog, forgetfulness, and anxiety. I still have a dry irritating sore throat & I hyperventilate especially after simple household tasks.

**Respondent 7:** in order of chronological appearance - Day one, generalized malaise and fever.

Day two, loss of smell and taste severe muscle pain; Day three, severe headaches.

Although I had lost my sense of smell and taste surprisingly, I still had an increased appetite. By the third day, my fevers had resolved (but I was on 1g paracetamol 4 hourly).

Day 5 to day 10, I experienced very severe acidity and heartburn. Today is exactly 3 weeks since I got my first symptom; for the past three days I have noticed insomnia. Although most of the symptoms have now resolved, I get occasional nasal stuffiness and headaches. And sense of taste and smell have also returned thankfully, although not yet to the previous levels. (But at least now I can tell chalk from cheese).

**Respondent 8:** generalized body aches, eye pain and feeling sleepy, loss of smell and taste, dull headache, fever and chills, excessive fatigue and breathlessness finally came the irritating coughs.

**Respondent 9:** extreme fatigue, slight flue but with lots of sneezing, cough that never gets productive, loss of smell, loss of taste, headache. One day I felt like my nostrils were burning when I would breathe in.

**Respondent 10:** I think the onset (before PCR test) my mouth felt acidic, that made me to brush my teeth several times that day. What followed was a sore throat and severe lower back ache. My tongue felt acidic but I could still taste and smell stuff. I got dry lips for a day or two. My complete blood and malaria checks were normal. Despite these, I had started myself on Azithromycin and Cold Pap, dexamethasone, but there was no change at all. Things just kept coming. Then I tested positive after 4 days, I started getting headaches and general tiredness/muscle aches. Day 2 after testing positive, I completely lost taste and smell. Day 3 I slept for 14 hours From Day 2–7 I had a running tummy. On day 4 I got high fevers 39–40°C, severe sweating, my face was dripping water like I was steaming (this caused me to sleep on the floor one of those days) I felt a fire burning in my feet. Breathing in was very bad for my throat; I felt like I was passing out through the throat got dizziness all through (my head kept spinning even in week 4) I got severe coldness (that chill you get when you stand near an open fridge) this I still have till now (week 4). For the first 2–3, I was totally disoriented. I could not fix my mind on anything, not even a simple assignment. Week 2, I got severe insomnia, and forgetfulness while in week 3, I became swollen (plump in my face and tummy) with blotting for 3 days. From week 4, I have experienced dry skin and recurrence of mild to moderate headaches. The sense for taste and smell are not fully back. I tested negative PCR on day 10.

As it is clear from these submissions, the symptoms vary, but are fairly troubling. I have noticed that fever as a symptom is not very common, calling into question the screening using thermo-guns that many institutions have implemented as part of COVID containment at entrances of buildings, and institutions. To people who have suffered from COVID-19, and have recovered, or at the very least tested negative on PCR (mine came on day 21, the day 14 test was still positive); my message is that let us be grateful to God for giving us another chance. The truth is we only tested negative, but let's temper that with reality. The bigger battle now starts. The risk of re-infection has not been quantified. So wear that mask, wash hands often and keep safe distance. You don't have to attend all parties you are invited to. We all who have now tested negative need to hope and pray that residual COVID antibodies don't slowly destroy our myelin sheaths of nerves or cause varying forms of delayed auto immune phenomena. We need to pray against the long-term effects that have been observed in other settings such as; lung fibrosis or chronic chest pain and lung disease or unexplained memory losses and strokes, insulin resistance and early onset hypertension, depression, mood instability and insomnia. These antibodies may fail to immunise you against another bout of COVID. Let's take it one day at a time. For our dear ones still in the struggle on the way to a negative result, be strong, don't give up... And take isolation in good stead, it's not just your earthly doctor's prescription, it is a prescription from God our Father Himself, saying to us in **Isaiah 26:20** --“Go, my people, enter your rooms and close your doors behind you. Hide for a little while until the wrath of God has passed.” It is indeed projected that this COVID- 19 pandemic could create overwhelming health and social economic strain in Africa and the rest of the world in the months to come^8^–^9^. This projection is based on the fact that Africa has inadequacies in the health systems, a weak health infrastructure, insufficient supplies of medicines, inefficient systems for surveillance and an overall ill-equipped laboratory capacity and scanty health personnel to optimally handle the pandemic^8^–^9^. Africa will especially have to device home grown solutions, and rapidly improve the number of trained health care workers and improve health delivery capacity to enable the continent to deal with the COVID-19 pandemic. Each outbreak does provide an opportunity to learn important information, to build capacity, train health care staff and to equip health institutions to handle current and future outbreaks or pandemics.
